# Prevalence of low back pain and associated factors among bank workers at Hawassa city, Northern Zone, Sidama Region, Southern Ethiopia

**DOI:** 10.1186/s12891-024-07594-9

**Published:** 2024-06-18

**Authors:** Thomas Jonga, Beniyam Samuel, Amdehiwot Aynalem, Eskinder Israel, Bargude Balta, Abdella Amano

**Affiliations:** 1 Hawassa University Comprehensive Specialized Teaching Hospital, Hawassa, Ethiopia; 2 Department of Midwifery, College of Medicine and Health Science, Dilla University, Dilla, Ethiopia; 3 School of Nursing, College of Medicine and Health Science, Hawassa University, Hawassa, Ethiopia; 4 Department of Reproductive Health, School of Public Health, College of Medicine and Health Science, Wolaita Sodo University, Wolaita Sodo, Wolaita Sodo, Ethiopia; 5 Hawassa University Comprehensive Specialized and Teaching Hospital, Hawassa, Ethiopia; 6 School of Public Health, College of Medicine and Health Science, Hawassa University, Hawassa, Ethiopia

**Keywords:** Low back pain, Bank workers, Prevalence, Associated factors, Hawassa, Ethiopia

## Abstract

**Background:**

Lower back pain (LBP) is a public health threat that affects people who frequently experience sedentary working conditions. Few studies reported on low back pain among bank workers in Ethiopia, particularly in the study area. Therefore, this study aimed to determine the magnitude and associated factors of low back pain among bank workers in Hawassa City, Sidama Region, Ethiopia.

**Methods:**

This institutional-based cross-sectional study was conducted from April 20, 2023, to June 30, 2023. A multistage sampling technique was employed to select participants, and data were collected using a structured self-administered questionnaire, entered into Epidata version 4.6, and transferred to SPSS version 25 for further analysis. Bivariate and multivariate logistic regression analyses were performed to identify the independent predictors of lower back pain.

**Results:**

Of the 627 total study participants, six hundred seven bank workers participated in the study, with a response rate of 96.8%. The overall magnitude of lower back pain among the study population was 55.2%, with a 95% confidence interval (CI 51.1–59). Based on the multivariate binary logistic regression analysis, being in a manager position (AOR = 3.85; 95% CI = (1.2,12), a level 2 banker (AOR = 3.8; 95% CI = (1.9,8.9), age 30–39 years (AOR = 4; 95% CI = (2,12.4), an age ≥ 40 years (AOR = 5.4; 95% CI= (3.04,16.3), working in sufficient space (AOR = 0.4; 95% CI = (0.3,0.9), and physical activity (AOR = 0.2; 95% CI = (0.1,0.8)) were significantly associated with low back pain.

**Conclusion:**

The prevalence of low back pain among the bank workers was high in the study area. Being in a managerial position, being a level two banker, being aged 30–39 years, being aged ≥ 40 years, working in sufficient space, and engaging in physical activity were significantly associated. Therefore, it is essential to establish a health screening team, create awareness programs for the benefit of physical activity, provide sufficient working space, and give special attention to elderly and senior bankers and bank managers to reduce the risk of developing low back pain.

## Background

Low back pain (LBP) is defined as short or persistent pain of the lower spinal cord, especially in the lumbar and sacral regions, and sometimes radiates toward the buttocks and lower legs. It is related to work-linked and uncomfortable postures [1]. LBP is a global public health concern that affects people who are frequently enrolled in sedentary working conditions and is a very common problem of the musculoskeletal system associated with working conditions [2, 3].

Occupation-associated factors are inseparable from low back pain. Any sedentary work that takes a long time, high workload, or irregular sitting preparation are the existing reasons for LBP [4]. The working style of bank workers is most commonly stagnant, and the alignment of their boards, computers, and tables is not adjusted or designed according to their health condition [5]. LBP among office workers, including bankers, leads to a negative economic impact, which including an increased absence from work and lost productivity, and has global implications for various sectors, such as economic, societal, and public health [6, 7].

LBP is a significant global public health concern affecting millions of people across different region. According to a 2016 assessment of the global burden of diseases, injuries, and risk factors, low back pain (LBP) was among the top 328 morbidities for any population category [8]. Globally, several studies have shown that office workers have a 1-month incidence of LBP, ranging from 23 to 46% [9, 10]. According to a World Health Organization report, in 2020, LBP had a considerable impact on 619 million people, with projections indicating a further increase to 843 million cases by 2050. It has been suggested that low-income countries have a higher frequency of LBP than high-income countries [9]. In Africa, a systematic review of population-based studies among children, adolescents, and adults estimated the lifetime (47%), annual (57%), and point (39%) prevalence of LB [11]. A study conducted in Dhaka, Bangladesh and Kigali, Rwanda, revealed that the prevalence of low back pain among bank workers was 36.6% and 45.8%, respectively [12, 13]. In Ethiopia, the point and twelve-month prevalence of LBP among the working population are 49% and 56%, respectively [14], and studies conducted in Addis Abeba, Jimma, and Gonder, Ethiopia, have shown that the prevalence of low back pain was 54.3%, 54%, and 55.4%, respectively [15–17].

Age, smoking, weight lifting, stooping, prolonged sitting, poor fitness, sedentary lifestyle, awkward posture at work, work experience, obesity, occupational stress, working conditions, long office hours, working in the same posture, and continuing the same job for many years were identified as the factors most strongly associated with LBP [12, 17–19].

The health problems associated with quality of life, disability, and economic influence as a result of health care expenses require ongoing public health interventions [3, 20, 21]. Determining the prevalence of low back pain provides a foundation for targeted interventions, research advancements, and improved well-being of bank workers. It also helps researchers prioritize studies related to low back pain, the development of evidence-based practices, and therapists in designing exercise programs, ergonomic adjustments, and stress management techniques to effectively address low back pain effectively and educate bank workers on preventive measures, emphasizing the importance of physical activity, stress reduction, and maintaining a healthy lifestyle. However, in Ethiopia, particularly in the study area, there are limited studies on the magnitude of LBP that can help establish planning strategies for the prevention and control of LBP among bankers. Therefore, this study aimed to determine the magnitude of low back pain and its associated factors among bank workers in Hawassa City, Sidama Region, Ethiopia.

## Methods and materials

### Study design

This cross-sectional study conducted from April 20, 2023, to June 30, 2023, among bank workers in Hawassa City, which is located in the Great Rift Valley, 275 km south of Addis Ababa. The city serves as the capital of Sidama National Regional State. According to 2016 Ethiopian Central Statistics Agency (CSA) data, the city had a population of 351,469. The city is home to many institutions that serve businesses and provide public services. There is one public bank with 31 branches a nd 42 private banks comprising 2669 bank workers.

### Population, sample size determination, and sampling techniques

All bank workers and bank workers who are in the selected banks in Hawassa City were the source and study population, respectively.

The survey included all Hawassa City bank employees who had been employed for a minimum of six months. A female employee or pregnant woman working at a bank with a child under six months of age, a previous surgical history of pelvic surgery, spinal surgery, LUCS, and other procedures; any history of back pain or other spinal injuries resulting from accidents (such as car accidents); a history of protrusion of the lumbar intervertebral disc, joint or bone disorders, or any history of persistent inflammatory pain (such as rheumatoid arthritis, ankylosing spondylitis, etc.); and bank workers with previous trauma were excluded from the study.

The sample size was determined using a single population proportion formula by considering the proportion of LBP among bank workers in Gondar City [17], which was approximately 55.4%, with a 95% confidence interval and a 5% margin of error. The final sample size was 627, after using a design effect of 1.5 and a 10% nonresponse rate.

### Sampling technique and procedure

A multi-stage sampling technique was used in this study. In the northern zone of the Sidaama region, Ethiopia, there were three city administrations; of the three city administrations, Hawassa City was randomly selected. There are eight sub-cities in Hawassa City; four of them—Menariya, Tabor, and Mehali sub-cities—were selected at random by the lottery technique. The number of banks in each sub-city was then proportionally calculated. Study participants were assigned to each bank using probability proportional to size (PPS) and were chosen using a simple random sampling method.

### Data collection instruments and procedures

Data were collected using structured, pretested, self-administered questionnaires adopted from the Nordic Musculoskeletal System Disorder [22] and adapted from similar studies performed previously on low back pain among bank workers. The questionnaires prepared in English language - were translated into the local language (Amharic) and retranslated back into English to check the consistency of their meaning. The data were collected via the Amharic version of the questionnaire by one BSc in Environmental Health and one BSc nurse and supervised by one MPH in Public Health who has experienced similar studies. One-day training was given to the data collectors and supervisors about the aim of the research, the data collection technique, ethical issues, and the content of the research in an understandable way. Every day, each completed questionnaire was checked for completeness, and the principal investigator controlled the overall data collection process.

### Data quality assurance

The questionnaire was prepared in English, then translated into Amharic, and finally retranslated back into English by independent translators to check for consistency. A pre-test was conducted on 5% of the sample size at the Alamura and Gebriel branches of the Commercial Bank of Ethiopia (CBE), which were not selected for the study. The supervisor reviewed the questionnaires daily to ensure uniformity, completeness, consistency, and missing data. The principal investigator held a brief meeting with the supervisor each day to verify the correct data collection before data collection was completed.

### Operational definition

#### Low back pain

A ba nk worker who had a perceived ache, pain, or discomfort localized below the coastal margin and above the inferior gluteal fold during the last 12 months was considered to have LBP [17].

#### Bank workers

employees who perform financial activities that include supervision, customer service, public relations, accounting clerks, loan officers, and managers [16].

#### Exercise

performing physical activity or performing any kind of sports activity, including walking, for at least 150 min per week [23].

#### Smoking cigarettes

Workers who had daily experience smoking approximately 1–4 cigarettes per day were considered light smokers, and those who smoked > 4 cigarettes were considered heavy smokers [24].

#### Alcohol consumption

Bank workers who are legal of drinking age and who drink two or more drinks that contain alcohol per day are considered to consume alcohol [25].

### Statistical analysis

Data were entered, cleaned, coded into EpiData version 4.6, and transferred to SPSS version 25 for further analysis. Descriptive statistics such as frequencies, percentages, and interquartile ranges were computed. Bivariate and multivariate logistic regression models were used to determine the degree of association between outcome and predictor variables. Independent variables with a *P* value less than 0.25 in the bivariable analysis were selected for multivariable logistic regression analysis. Multivariable logistic regression analysis was performed to identify statistically significant variables. Statistical significance was set at a *P*-value of less than 0.05. The model’s fitness was checked using Hosmer and Lemeshow’s goodness- of- fit test, and the results were considered significant at a *p*-value greater than 0.05. The variance inflation factor (VIF) was used to evaluate the potential for multicollinearity among independent variables.

## Results

### Sociodemographic characteristics of the participants

A total of 627 bank workers participated in the study, with a 96.78% response rate. Of the 607 respondents, 315 (51.9%) were male. The mean age of the participants was 32.85 ± 6.8 years. Of the total participants, 507 (83.5%) were under the age of 40 and 491 (80.9%) were in the Level 1 banker position (Table 1).

**Table 1:** Sociodemographic characteristics of bank workers in Hawassa City, Northern Zone, Sidama Region, Ethiopia, 2023 (*n* = 607)

Variables	Response category	Frequency (*N* = 607)	Percent (100%)
Age	20–29	100	37.7
	30–39	278	45.8
	≥ 40	229	16.5
Sex	Male	315	51.9
	Female	292	48.1
Educational status	Diploma	50	8.2
	BA	496	81.7
	MSc or above	61	10
Marital status	Married	476	78.4
	Single	79	13.1
	Divorced	44	7.2
	Widowed	8	1.3
Job designation	Manager	43	7.1
	Level 1 banker	491	80.9
	Level 2 banker	73	12
Income	8,000–16,000	275	45.3
	16,000–32,000	212	34.9
	≥ 32,000	120	19.8
Experience in bank	< 10 years	332	54.7
	10–15 years	267	44
	≥ 15 years	8	1.3
BMI	< 18.5	38	6.3
	18.5–24.9	285	46.9
	25-29.9	279	46
	≥ 30	5	0.8

### Work-related characteristics

More than two-thirds of the study participants 427(70.3%) had 8–12 working hours, and slightly more than half of the study participants 348(57.3%) had no training in workplace safety. Most participants 548(90.3%) used computers for their daily activities, and approximately two-thirds 382(62.9%) used computers for ≥ 6 h. Majority of the study participants 418(69.9%) had no ergonomic training. Half of the participants 324(53.3%) reported that they had insufficient space to properly perform their work (Table 2).

**Table 2:** Work-Related Characteristics of Bank Workers in Hawassa City, Northern Zone, Sidama Region, Ethiopia, 2023 (*n* = 607)

Variables	Response category	Frequency (*n* = 607)	Percent
Working hours	< 8	12	27.7
	8–12	427	70.3
	≥ 12	168	2
Training on workplace safety	Yes	259	42.7
	No	348	57.3
Training on Ergonomics	Yes	189	30.9
	No	418	69.1
Sufficient space to do your work properly	Yes	283	46.7
	No	324	53.3
Rest break (tea break) in between the shift	Yes	232	38.2
	No	375	61.8
Involve in lifting or carrying heavy objects	Yes	186	30.6
	No		69.4
ours do you sit before rest time	3	129	21.2
	3–4	284	46.8
	≥ 4	194	32
Use computer	Yes	548	90.3
	No	59	9.7
How long do you use the computer per day	< 6 h	225	37.1
	≥ 6 h	382	62.9
Type of chair	With armrest	196	32.3
	Without armrest	411	67.7
Chronic disease	Yes	5	0.8
	No	602	99.2
Bending/twisting sitting posture while you perform different tasks	Yes	279	46
	No	328	

### Behavior-related characteristics

More than two-thirds of the participants 427(70.3%) and 413(68.5%) had no alcohol consumption and smoking history, respectively. habit Most of the participants had 383 (63.1%) physical activities; of these, the fourth 284(74.2%) performed < 150 min (Table 3).

**Table 3:** Behavioral-Related Characteristics of Bank Workers in Hawassa City, Northern Zone, Sidama Region, Ethiopia, 2023 (*n* = 607)

Variables	Response category	Frequency (*n* = 607)	Percent (100%)
Alcohol use	Yes	180	29.7
	No	427	70.3
Smoking cigarette currently	Yes	190	31.5
	No	413	68.5
How many cigarettes	1–4	113	59.5
	≥ 4	77	40.5
Physical activity	Yes	383	63.1
	No	224	36.9
How many minutes/weeks of physical activity	< 150	284	74.2
	≥ 150	99	25.8

### The magnitude of lower back pain

According to this study, the prevalence of the bank employees’ 12-month LBP was 55.2%, with a 95% confidence interval (CI 51.1–59). Because of their LBP, nearly three-fourths (69.9%) of the respondents did not quit their jobs, 38.8% claimed that standing for extended periods worsened their pain, and 67.1% of study participants found that resting helped them feel better (Table 4).

**Table 4:** Low Back Pain-Related Characteristics of Bank Workers in Hawassa City, Northern Zone, Sidama Region, Ethiopia, 2023 (*n* = 335)

Variables	Response category	Frequency (*n* = 335)	percent %
Low Back Pain during the last 12 months	Yes	335	55.2
	No	272	44.8
Absence from work due to LBP	Yes	101	30.1
	No	234	69.9
Pain worsening factor at work	Prolong sitting	71	21.2
	Prolong standing	130	38.8
	Prolong bending	36	10.7
	Twisting movements	98	29.3
The time when the pain worsens	At work	210	62.7
	At home	110	32.8
	Every time	15	4.5
Pain relieved by	Pain killers	72	21.5
	Rest	225	67.1
	None	38	11.4

### Factors associated with LBP

According to the binary logistic regression, monthly income, education level, job position, work hours per day, training on workplace safety, sufficient work space, training on ergometric issues, and body mass index (BMI) were factors associated with LBP. However, being in a managerial position, being a level 2 banker, being aged 30–39 years and ≥ 40 years, work space sufficiency, and physical activity were significantly associated factors in the multivariable logistic regression analysis. The odds of LBP were 3.9 times greater for bank workers who were in a managerial position than for bank workers who were not (AOR = 3.85; 95% CI = 1.2, 12). Bankers aged 30–39 and ≥ 40 years had 4- and 5-fold greater odds of having LBP, respectively, than bankers aged 20–29 years (AOR = 4; 95% CI = 2,12.4 and AOR = 5.4; 95% CI = 3.04,16.3, respectively). The odds of LBP were 80% lower for bank workers who performed regular physical activities than for their counterparts (AOR = 0.2, 95% CI = 0.1–0.8). Bankers who worked in a sufficient space were 60% less likely to develop LBP than those who worked in an insufficient space (AOR = 0.4; 95% CI = 0.3–0.9). The odds of LBP were 3.8 times greater for bank workers who were at level two than for those who were at level one (AOR = 3.8; 95% CI = (1.9, 8.9)) (Table 5).

**Table 5:** Bivariate and multivariate analyses of factors associated with LBP among bank workers in Hawassa City, Northern Zone, Sidama Region, Ethiopia, 2023 (*n* = 607)

Variables		Low back pain(LBP)				Odds Ratio (95% CI)		
		Yes (%)	No (%)			Bivariate(COR)	Multivariate(AOR)	
Monthly income								
8000–16,000		164(59.6)	111 (40.4)			1.7(1.5,2.4)	1.3(0.4,1.6)	
16,000–32,000		115(54.3)	97 (45.7)			1.4(0.36,1.6)	0.5(0.6,1.5)	
≥ 32,000		56 (46.7)	64(53.3)			1	1	
Education level								
Diploma		4 [8]	46(92)			0.3(0.14,0.5)	0.03(0.07,0.12)	
Degree		274(55.2)	222(44.8)			3.8(1.22, 6.2)	3.3(0.1,5.4)	
MSc and above		15(24.6)	46(75.4)			1	1	
Job position								
Manager		21(48.8)	22(51.2)			4.9 (2.25,12.6)	3.85(1.2,12) * *	
Level 2 Banker		253(51.5)	238(48.5)			4.8(2.5,9)	3.78(1.9,8.5) *	
Level 1 Banker		12(16.5)	61(83.5)			1.00	1.00	
Work hours per day								
≥ 12		58(34.5)	110(65.5)		2.6(1.47,4.14)		1.8(0.5, 3.43)	
8–12		160(37.5)	267(62.5)		2.9 (1.7, 4.94)		1.9(0.53, 3.97)	
< 8 h		2(16.6)	10(83.3)		1		1	
Training on workplace safety								
Yes		56(21.6)	203(78.4)		0.5(0.16,0.93)		0.4(0.3,1.8)	
No		132(38)	216(62)		1		1	
Workspace sufficiency								
Yes	60 (21.2)		223 (78.8)		0.51(0.1,0.2)		0.4(0.3,0.9) * * *	
No	112(34.6)		212 (65.4)		1		1	
Training on ergometric issues								
Yes		26(13.8)	163(86.2)			0.2(0.04,0.8)	0.3(0.1,1.1)	
No		170(40.7)	248(59.3)			1	1	
Age								
20–29		90(90)		10 [10]		1	1	
30–39		139(50)		139(50)		9 (4.2,15)	4.2(2,12.4) *	
> 40		106(46.3)		123(53.7)		10(7.4,22)	5.4(3.04,16.3) * *	
Physical activity								
Yes		112(29.2)		271(70.8)		0.5(0.1,0.8)	0.2(0.1,0.8) *	
No		101(45.1)		123(54.9)		1	1	

## Discussion

This study assessed the prevalence of low back pain and its associated factors among bank workers in Hawassa City, Sidama Region, Ethiopia.

This study found that a substantial proportion of bank employees (55.2%) were affected by LBP. This finding was supported by the 95% confidence interval, which ranged from 51.1% to 59%. This indicates that back pain is common among bank employees in the study area. The high prevalence of low back pain among bank workers in this study could result in problems with day-to-day activities and performance at work, a significant economic burden, and the cause of personal hardship. It also reduces the productivity of employees by challenging them to perform their tasks efficiently, resulting in frequent absences that can disrupt workflow and impact overall team performance; workforce absenteeism affects staffing levels, customer service, and operational continuity, affecting the quality of customer service by making it more difficult for bank employees to maintain a positive demeanor, answering queries, and providing efficient service and costs for healthcare, resulting in stress, anxiety, frustration, and affecting their overall well-being.

Our findings are nearly consistent with those of previous studies performed in Gonder, Northern Ethiopia (55.4% [17], Addis Ababa city (54.3% [16], and Jimma town (54.4% [15]. However, it is higher than the findings other studies conducted in developed and developing countries. For instance, (India, 40.4% [26]; Dhaka city, in Bangladesh, 36.6% [12]; Benta, in South Africa, 47% [7]; Kuwait, western Asia, 51.1% [27]; Southwest Nigeria, 38% [28]; Kigali Rwanda, 45.8% [13]; Mekelle, 40.3% [29]; and Wolaita, southern Ethiopia, 38.4% [30], and lower than the results of studies performed in Saudi Arabia, 73% [31]; and Mekelle, 74.8% [32]. The reason for this variation might be due to differences in sample size, study population and setting, and classification of LBP. This could also be due to socio-demographic and geographic discrepancies.

.

According to our study, bank employees aged between 30 and 39 years and those aged ≥ 40 years were more likely to experience back pain than employees aged between 20 and 29 years. This finding aligns with a previous study in Dhaka, Bangladesh [12], which revealed that people older than 40 years had a greater risk of lower back pain than those in younger age groups. Another study in the United States highlighted the association between advanced age and low back pain. This may be because older age is a major risk factor for LBP [26]. Another reason could be that, as workers age, they may experience increased susceptibility to LBP due to factors such as reduced muscle strength, flexibility, and degenerative changes in the spine.

Additionally, this study demonstrated that physically active bankers had a lower likelihood of experiencing lower back pain than those who did not. This result is consistent with a study conducted in Dhaka, Bangladesh [12], which may be because the spine can become misaligned due to weak or shortened muscles; on the other hand, regular exercise can strengthen the muscles that support and maitain the spine in ideal alignment for optimal performance [27]. Another cause could be that their inactivity increases their chance of having low back pain (LBP) by causing weakness of muscles in the legs, pelvis, and back [28]. A sedentary lifestyle that causes a lack of physical exercise impairs muscular strength, power, and the capacity of the spinal disc to hold water at a normal concentration [29]. The level of physical activity should be appropriate for the worker’s age. According to certain studies, women over the age of 65 who engage in vigorous (at least 20 min of intense physical activity on three or more days per week) or moderate (at least 30 min of moderate- intensity activity on five or more days per week) physical activity are at a significantly greater risk of developing persistent low back pain. After controlling for age and body mass index (BMI), walking for thirty minutes five days a week, and strength training twice a week can reduce the incidence of chronic low back pain (LBP) [30].

According to the current study, bank employees who hold level two job positions and managerial positions are more likely to experience lower back discomfort than those who do not. This could be because bank managers work longer hours, which could be a significant factor contributing to LBP. The associations between being in a managerial position or at the banker level and lower back pain have not been studied. On the other hand, some studies indicate that working long hours at an office increases the risk of low back pain. A study from Bangladesh [12] and Denmark [31], for example, showed that employees who worked longer hours in an office had a greater incidence of low back pain (LBP) than did those who worked fewer hours in a sedentary environment.

Our study also indicates that bank employees who work in a sufficient space have a decreased likelihood of developing LBP compared to those who do not. This can be the result of a decrease in mobility and preoccupation, which leads to back pain due to extended sitting. Working conditions are frequently assumed toplay a significant role in the development of back pain [32]. Working in a bent and twisted position for extended periods, bending significantly with the trunk, and performing repetitive activities with the trunk are among the postures linked to it [33–35]. One study, for instance, from Kigali, Rwanda [13], found that a certain f sitting position could predict the occurrence of back discomfort. Compared to bank employees who sat with their backs straight, those who sat with their backs bent were more likely experience back pain. Similarly, bank employees who used to sit with their backs twisted were more likely to experience back pain than were those who did not. This bad posture can compress and stiffen the lower back, which can cause harm. However, working in a sufficiently spacious environment improves bank workers’ well being. For instance, providing ample space for bank workers can lead to better posture and reduce strain on the lower back. This, in turn, may contribute to less low back pain, overall well-being, job satisfaction, reduced healthcare costs for both employees and employers, and decreased absenteeism which translates to financial savings for the organization. When employees experience less pain and discomfort, they are likely to be more productive. Working in comfortable conditions can positively impact focus, concentration, and efficiency and could result in fewer sick days or medical leaves taken by employees due to discomfort or pain. These implications would need to be supported by research and evidence to ensure working in a sufficiently spacious environment plays a role in LBP prevention.

### Study limitations

The current study had several limitations. The main limitation of this study was inability to establish a causative relationship between exposure and LBP because of the nature of the study design. The study was conducted through the collection of subjective data via self-administeredquestionnaires, which may be vulnerable to reporting bias due to the respondent’sinterpretation of the questions or their inclination to express their emotions. It is possible that the subjects were unwilling to state the truth or that they had trouble recalling specific details of events or traits. This is because people with LBP do not live a typical life, which lowers their self-esteem. Despite these limitations, this study has important implications for public health, and this information may help develop intervention plans aimed at reducing back pain and its associated risk factors.

### Conclusions and recommendations

The magnitude of low back pain among the bank workers was high in the study area. Being in a managerial job position, being a level 2 banker, advanced age, workspace sufficiency, and physical activity were significant factors associated with lower back pain. To minimize the effects on the economy, society, and public health as well as psychological distress, pain catastrophizing, fear of movement, low self-confidence to overcome, poor expectations for recovery, perceptions of greater functional loss, and pain-related challenges, immediate health intervention is needed. It would be preferable to focus on a safe working environment and routine health screening programs for bank employees. It is better to pay special attention toelderly bankers and bank managers. A bank worker who has developed low back pain should consult with healthcare professionals for personal advice. Providing ergonomic workstations and promoting physical activity can mitigate the impact of LBP on bank workers’ practices.

## Data Availability

On reasonable request, the corresponding author will provide the complete data set and additional study-related information.

## Abbreviations

AOR: Adjusted Odds Ratio
BMI: Body Mass Index
CI: Confidence Interval
LBP: Low Back Pain
LUCS: Lower Uterine Segment, Section, MSDs, Musculoskeletal Disorders
SPSS: Statistical Package for Social Sciences
WHO: World Health Organization

## References

[1] Hoy D, Brooks P, Blyth F, Buchbinder R. Best Practice & Research Clinical Rheumatology The Epidemiology of low back pain. Best Pract Res Clin Rheumatol. 2010;24(6):769–81. 10.1016/j.berh.2010.10.002. 10.1016/j.berh.2010.10.002 21665125

[2] Great Britain. 2021. Work-related musculoskeletal disorders statistics in Great Britain,. 2021;(December).

[3] Clark S, Horton R. Comment Low back pain: a major global challenge. Lancet. 2018;6736(18):30725. 10.1016/S0140-6736(18)30725-6. 10.1016/S0140-6736(18)30725-6 29573869

[4] Ye S, Jing Q, Wei C, Lu J. Risk factors of non-specific neck pain and low back pain in computer- using office workers in China: a cross- sectional study. BMJ Open. 2017;7(e014914):9–11. Available from: 10.1136/bmjopen-2016-014914. 10.1136/bmjopen-2016-014914 PMC5594207 28404613

[5] Singh K, Haile GG (2018). Associated factors of low back Pain among the Workers of Commercial Bank of Ethiopia. J Med Sci Technol.

[6] WHO G. WHO_TRS_714.pdf.

[7] Naude B. Factors Associated with Low Back Pain in Hospital Employees. 2008.

[8] Metrics GH, Global. regional, and national incidence, prevalence, and years lived with disability for 328 diseases and injuries for 195 countries, 1990–2016: a systematic analysis for the Global Burden of Disease Study 2016. 2017;390:1990–2016. 10.1016/S0140-6736(17)32154-2 PMC5605509 28919117

[9] Campos-Fumero A, Delclos GL, Douphrate DI, Felknor SA, Vargas-Prada S, Serra C et al. Low back pain among office workers in three Spanish-speaking countries: findings from the CUPID study. BMJ Inj Prev. 2017;23(3):158–64. https://injuryprevention.bmj.com/lookup/doi/10.1136/injuryprev-2016-042091. 10.1136/injuryprev-2016-042091 PMC5531253 27585564

[10] Shigeki MUTO, Takashi MUTO, Akihiko SEO, Tsutomu YOSHIDA, Kazushi TAODA. and MW. Preval Risk Factors Low Back Pain. 2006;123–7. 10.2486/indhealth.44.123 16610547

[11] Morris LD, Daniels KJ, Ganguli B, Louw QA. An update on the prevalence of low back pain in Africa: a systematic review and meta-analyses. BMC Musculoskelet Disord. 2018;19(196):2–15. https://bmcmusculoskeletdisord.biomedcentral.com/articles/10.1186/s12891-018-2075-x. 10.1186/s12891-018-2075-x PMC6055346 30037323

[12] Ali M, Ahsan GU, Hossain A. Prevalence and associated occupational factors of low back pain among the bank employees in Dhaka City. J Occup Health. 2020;62(1):1–10. https://academic.oup.com/joh/article/7249947. 10.1002/1348-9585.12131 PMC7383126 32715531

[13] Kanyenyeri L, Asiimwe B, Mochama M, Nyiligira J, Habtu M. Prevalence of Back Pain and Associated Factors among Bank Staff in Selected Banks in Kigali, Rwanda: A Cross Sectional Study. Heal Sci J. 2017;11(3):1–7. http://www.hsj.gr/medicine/prevalence-of-back-pain-and-associated-factors-among-bank-staff-in-selected-banks-in-kigali-rwanda-a-cross-sectional-study.php?aid=19571.

[14] Jegnie M, Afework M. Prevalence of Self-Reported Work-Related Lower Back Pain and Its Associated Factors in Ethiopia: A Systematic Review and Meta-Analysis. Gualano MR, editor. J Environ Public Health. 2021;2021:1–19. https://www.hindawi.com/journals/jeph/2021/6633271/. 10.1155/2021/6633271 PMC8486508 34603457

[15] Etana G, Ayele M, Gerbi A. DA. Prevalence of work related Musculoskeletal disorders and Associated Factors among Bank Staff in Jimma City, Southwest Ethiopia, 2019: an Institution-based cross-sectional study. Dovepress/Journal Pain Res. 2021;(December 2020). 10.2147/JPR.S299680 PMC8275204 34267551

[16] Dagne D, Abebe SM, Getachew A. Work-related musculoskeletal disorders and associated factors among bank workers in Addis Ababa, Ethiopia: a cross-sectional study. BMC Environ Heal Prev Med. 2020;25(1):33. 10.1186/s12199-020-00866-5. 10.1186/s12199-020-00866-5 PMC7385884 32718332

[17] Workneh BS, Mekonen EG. Prevalence and Associated Factors of Low Back Pain Among Bank Workers in Gondar City, Northwest Ethiopia. Orthop Res Rev. 2021;13:25–33. https://www.dovepress.com/prevalence-and-associated-factors-of-low-back-pain-among-bank-workers--peer-reviewed-article-ORR. 10.2147/ORR.S300823 PMC7881777 33603503

[18] Fanta M, Alagaw A, Kejela G, Tunje A. Low back pain and associated factors among civil service sectors office workers in Southern Ethiopia. Int J Occup Saf Heal. 2020;10(1):53–63. https://www.nepjol.info/index.php/IJOSH/article/view/29883.

[19] Hanna F, Daas RN, El-Shareif TJ, Al-Marridi HH, Al-Rojoub ZM, Adegboye OA. The Relationship Between Sedentary Behavior, Back Pain, and Psychosocial Correlates Among University Employees. Front Public Heal. 2019;7(April):1–7. https://www.frontiersin.org/article/10.3389/fpubh.2019.00080/full. 10.3389/fpubh.2019.00080 PMC6465323 31024881

[20] Yitayeh A, Fasika S, Mekonnen S, Gizachew M. Work related musculoskeletal disorders and associated factors among nurses working in governmental health institutions of Gondar town, Ethiopia, 2013. Physiotherapy. 2015;101(1):e1694. https://linkinghub.elsevier.com/retrieve/pii/S0031940615001327.

[21] Tosunoz IK (2017). Low back Pain in nurses. Int J Caring Sci.

[22] Kuorinka I, Jonsson B, Kilbom A, Vinterberg H. Standardised Nordic Questionnaires Anal Musculoskelet Symptoms. 1987;233–7. 10.1016/0003-6870(87)90010-x 15676628

[23] WHO. Geneva 2010. Global recommendation on physical for health.

[24] Dr Kjell Bjartveit. Health consequences of smoking 1–4 cigarettes per day. 2005;20–4. 10.1136/tc.2005.011932 PMC1748107 16183982

[25] States U. Understanding Alcohol ’ s Adverse Impact on Health. 2022.

[26] Wong AY, Karppinen J, Samartzis D. Low back pain in older adults: risk factors, management options and future directions. Scoliosis Spinal Disord. 2017;12(1):14. http://scoliosisjournal.biomedcentral.com/articles/10.1186/s13013-017-0121-3. 10.1186/s13013-017-0121-3 PMC5395891 28435906

[27] Healy GN, Eakin EG, Owen N, Lamontagne AD, Moodie M, Winkler EAH et al. A Cluster Randomized Controlled Trial to Reduce Office Workers’ Sitting Time. Med Sci Sport Exerc. 2016;48(9):1787–97. https://journals.lww.com/00005768-201609000-00019. 10.1249/MSS.0000000000000972 27526175

[28] Warnakulasuriya SSP, Peiris-John RJ, Coggon D, Ntani G, Sathiakumar N, Wickremasinghe AR. Musculoskeletal pain in four occupational populations in Sri Lanka. Occup Med (Chic Ill). 2012;62(4):269–72. https://academic.oup.com/occmed/article-lookup/doi/10.1093/occmed/kqs057. 10.1093/occmed/kqs057 PMC3428920 22661663

[29] Andersen LB, Sci M, Wedderkopp N, Leboeuf-yde C (2006). Association between Back Pain Phys Fit Adolescents.

[30] Kim W, Jin YS, Lee CS, Hwang CJ, Lee SY, Chung SG et al. Relationship Between the Type and Amount of Physical Activity and Low Back Pain in Koreans Aged 50 Years and Older. PM&R. 2014;6(10):893–9. 10.1016/j.pmrj.2014.04.009. 10.1016/j.pmrj.2014.04.009 24780849

[31] Gupta N, Christiansen CS, Hallman DM, Korshøj M, Carneiro IG, Holtermann A. Is Objectively Measured Sitting Time Associated with Low Back Pain? A Cross-Sectional Investigation in the NOMAD study. Dorner TE, editor. PLoS One. 2015;10(3):e0121159. 10.1371/journal.pone.0121159. 10.1371/journal.pone.0121159 PMC4373888 25806808

[32] Koes BW, Bouter L. Preventing low back pain in industry - Reply. J Am Med Assoc. 2015;280(23). Available from: Jama.ama-assn.org.

[33] Karacan I, Aydin T, Sahin Z, Cidem M. Facet angles in lumbar disc herniation: their relation to Anthropometric features. 2004;29(10):1132–6. 10.1097/00007632-200405150-00016 15131443

[34] Bener A, Alwash R, Tariq Gaber GL (2016). Obes Low Back Pain.

[35] Punnett L, Prüss-Ütün A, Nelson DI, Fingerhut MA, Leigh J, Tak S et al. Estimating the global burden of low back pain attributable to combined occupational exposures. Am J Ind Med. 2005;48(6):459–69. https://onlinelibrary.wiley.com/doi/10.1002/ajim.20232. 10.1002/ajim.20232 16299708

